# Primary Undifferentiated Pleomorphic Sarcoma of the Breast: A Case Report with Literature Review of Similar Cases

**DOI:** 10.30699/IJP.2023.2006411.3139

**Published:** 2023-12-29

**Authors:** Amirhossein Jafarian, Mohaddeseh Shahraki, Samaneh Sajjadi, Behrooz Daneshmand

**Affiliations:** 1 *Cancer Molecular Research Center* *, Mashhad University of Medical Sciences, Mashhad, Iran*; 2 *Department of Pathology, Faculty of Medicine, Mashhad University of Medical Sciences, Mashhad, Iran*; 3 *Department of Internal Medicine, Faculty of Medicine, Mashhad University of Medical Sciences, Mashhad, Iran*

**Keywords:** Breast, Breast neoplasms, Mastectomysarcoma

## Abstract

Breast sarcoma is a rare but aggressive tumor. There are few case reports in the literature and several aspects of this disease are still not completely comprehended. Therefore, reporting new cases can help to enrich the literature.

We report a patient with breast mass and pus secretion from her right breast, misdiagnosed as an abscess and mistreated by antibiotics. The patient was referred for an ultrasound examination and mammography, and a needle biopsy was performed that suggested an aggressive tumor. By the pathologist's suggestion, a total mastectomy of the right breast was performed with the excision of sentinel nodes. A pathological examination revealed a high-grade undifferentiated pleomorphic sarcoma (UPS) without vascular or lymph node invasion as the final diagnosis. The patient underwent postoperative chemotherapy and is currently in good condition.

This case emphasizes considering this rare tumor when approaching a breast mass. Performing surgery with adequate resection margin can improve the patient's prognosis. Some suggested breast UPS cases with lung and brain metastasis would be more aggressive tumors than other breast sarcomas. Total mastectomy with negative margins and free-of-tumor lymph nodes may be the key to improve prognosis in such patients.

## Introduction

Breast sarcoma is a rare and heterogeneous group of non-epithelial tumors arising from the mesenchymal tissue of the breast. Most sarcoma cases of the breast are secondary to radiation therapy of breast tissue or other intrathoracic cancers, while primary cases are sporadic (<1% of all primary breast malignancies) (1, 2). It mainly presents with rapid-growing, firm, well-defined, unilateral mass, pain, or overlying skin changes in the fifth or sixth decades of life, commonly in females (97.6%) (2). The prognosis of the patients, like sarcomas of soft tissue in other organs, depends on the tumor size, depth, site, histological subtype, and patients' age (3). 

Undifferentiated pleomorphic sarcoma (UPS), previously defined as malignant fibrous histiocytoma, is a rare spindle cell neoplasm of the skin and soft tissue, classified into superficial and deep UPS, respectively; like other sarcomas, the prognosis is based on tumor's size, depth, and anatomical site (4, 5). Most cases occur in extremities, chest wall, retroperitoneum, head, and neck, while breast is not a common site for UPS. Considering the scarcity of cases presenting with breast UPS, diagnosis of this entity is challenging (6).

Here, we present a woman with a 2-month history of breast mass, misdiagnosed as an abscess and mistreated with antibiotics. An ultrasound examination and a needle biopsy provided suspicion of an invasive tumor; finally, the diagnosis was confirmed by a pathological examination of the surgically removed lesion (including breast and sentinel lymph nodes). We also performed a literature review on the cases published, which can provide a better perspective for the physicians and can help to improve the patient’s prognosis through early diagnosis and effective treatment.

## Case Presentation

**Fig. 1:**
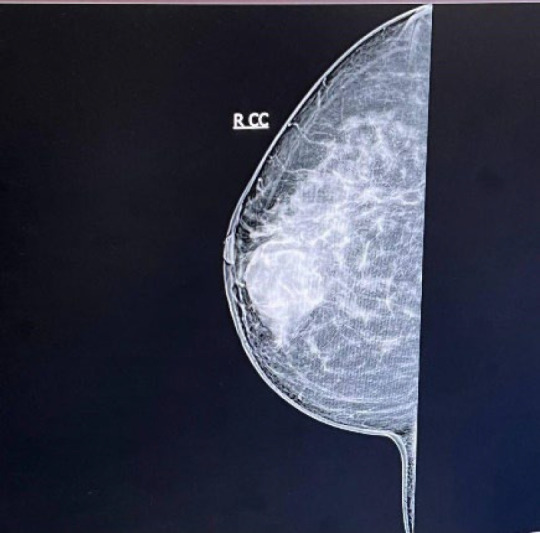
The results of the mammography examination of the right breast

**Fig. 2:**
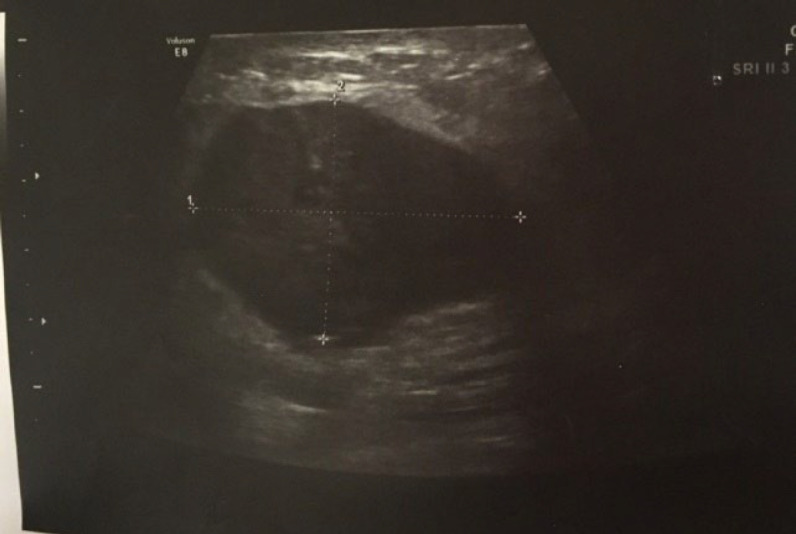
Ultrasound examination of the right breast

**Fig. 3:**
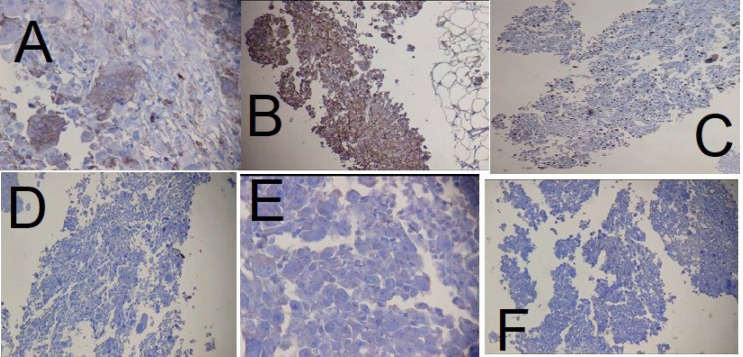
Immunohistochemical staining of the needle biopsy specimen showing positivity of the tumoral cells for A. CD68 (×400), B. Vimentin (×100), and C. Ki67 index was 50% (×100); but negative for D. CK (×100), E. ER (×400), and F. EMA (×100)

A 50-year-old woman was referred to us on 01 Dec 2022 at Mehr Hospital, Mashhad, Iran, with a 2-month history of heaviness, solidness, redness, pain, and pus secretion in the right breast that was treated by antibiotics with the assumption of abscess, which resolved the pus secretion but not the other symptoms; 1 month later, the patient felt that the mass has become larger and referred to our center. Physical examination by a physician showed erythema and mass in the superior interior part of the areola. The results of the serum test showed an increased white blood cell (11.6 ×10^3^ /µL; 82% neutrophil, 14% lymphocyte, and 4% eosinophil); other tests, including red blood cell, hemoglobin, hematocrit, platelet count, thyroid stimulating hormone, follicular stimulating hormone, prolactin, and beta-human chorionic gonadotropin were within normal range. A mammographic examination showed a heterogeneously dense composition that could obscure a small lesion. The mass was defined to have a 45-mm diameter with partially obscured margins in the posterior of the right breast's areola; increased density was observed in the superolateral area of the left breast (Figure 1). An ultrasound examination of the breast, performed by an expert radiologist, showed normal fibro-glandular tissue in the right breast with a well-circumscribed heterogenic hypoechoic mass, sized 42×32 mm in the superomedial part of the right areola, connected to the nipple at about 10 mm distance from the skin (Figure 2). The nipple and skin of the right breast were erythematous. A reactive lymph node with a maximum SAD of 5 mm was observed in the right axillary region. The radiologist suggested tissue diagnosis for a definite diagnosis. The left breast was normal in the ultrasound examination. The abdominal ultrasound also showed a normal uterus and ovaries with no col-de-sac fluid. Considering the results of the breast ultrasound, the physician performed the needle biopsy for the patient and sent the specimen to the pathologist. In the macroscopic examination, a 0.5-1 cm creamy specimen with a thickness of 0.2 cm was observed. The sections prepared from the specimen contained necrotic breast tissue with fat and mixed inflammatory infiltration, small foci of neoplastic lesions containing osteoclast-type giant cells, and scattered atypical epithelioid cells. Immunohistochemistry (IHC) on needle biopsy specimens showed positive vimentin and CD68 in the tumoral cells, 50% Ki67 labling index, and negative CK, CAM5/2, EMA, Melan A, HMB45, and ER (Figure 3). The pathologist suggested an invasive carcinoma with osteoclastic-like giant cells that could not be confirmed by this specimen, considering the negative epithelioid markers and the tumor might be another rare lesion, such as a giant cell tumor. The pathologist suggested total resection of the breast. 

The patient was scheduled for radical mastectomy of the right breast with excision of axillary lymph nodes; during surgery, the frozen specimens of the right breast with skin and sentinel lymph nodes were sent for the pathological examination. As the pathology reports free margins and no lymph node involvement, the surgery was ended, and the rest of the lymph nodes were not removed.

**Fig. 4:**
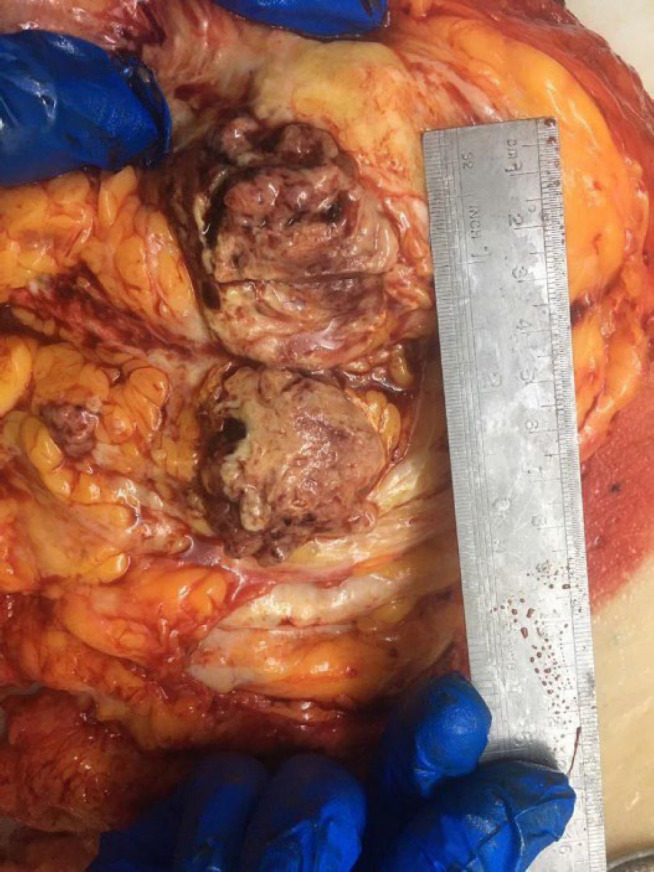
Macroscopic image of the right breast

**Fig. 5:**
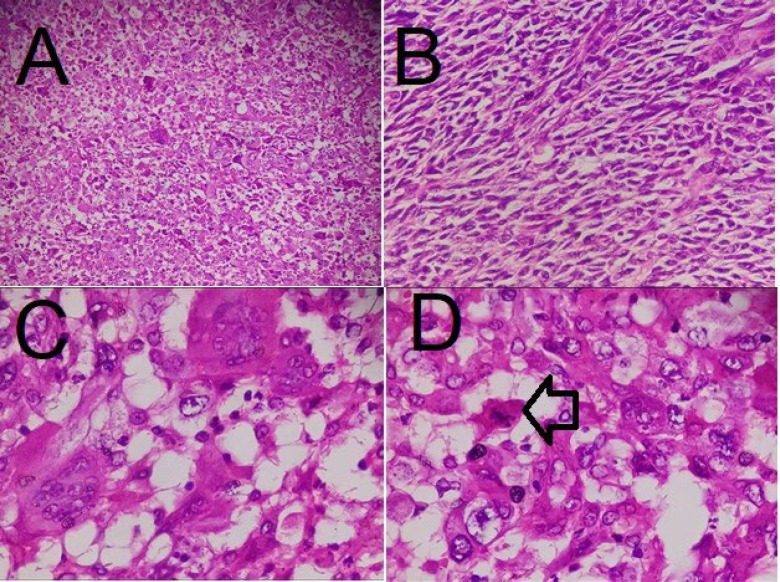
Microscopic images with hematoxylin and eosin staining showing atypical spindle and epithelial cells (A and B ×100) with osteoclastic-like giant cells, high mitotic rate, and pleomorphism (C and D ×400); the arrow indicates mitosis

**Fig. 6:**
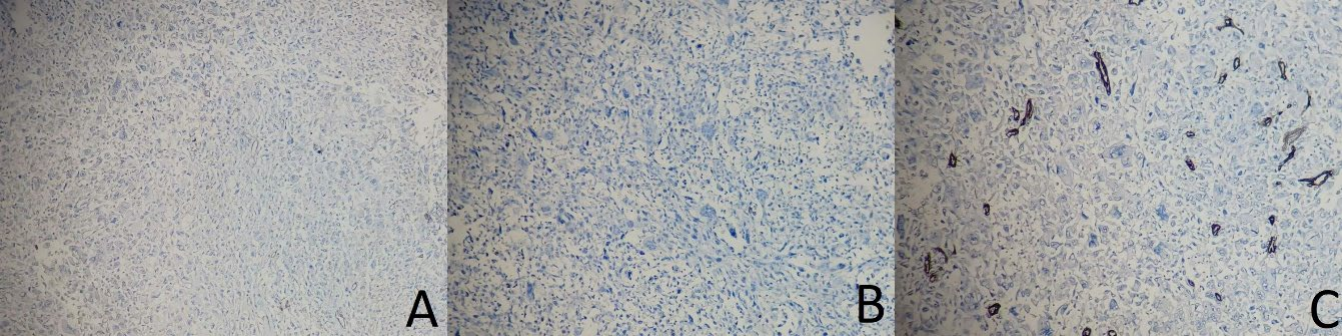
Immunohistochemical staining of the resected specimen showing negativity of t the tumoral cells for A. CK (×100), B. P63 (×100), and C. CD34 (×100)

An accurate pathological examination is shown in the figure. The macroscopic study showed the right breast (measuring 23×19×7.2 cm; Figure 4) with two fibro-adipose tissue fragments (total measuring 3.5×2.5×2 cm) that serial sectioning revealed two lymph nodes with a diameter of 0.5 and 2.5 cm. The serial coronal sectioning of the breast showed an ill-defined infiltrative soft, heterogeneous, and necrotic tumor bed in the center of the breast, measuring 6 × 5.5 × 4 cm, within 2 cm of the closest resection margin (deep margin). The microscopic evaluation showed a neoplastic proliferation of atypical spindle and epithelial cells with high mitotic rate, pleomorphism, and osteoclastic-like giant cells with atypical mitosis and necrosis (30%; Figure 5). According to the previous immunohistochemical results and microscopic appearance, the final diagnosis was high-grade UPS (4/4) without vascular or lymph node invasion. We also performed IHC on the resected specimen on separate blocks, which showed negative CK, P63, and CD34 (Figure 6; A, B, and C, respectively).

The patien underwent adjuvant chemotherapy and was in good condition in the last follow-up (2 months after the surgery).

## Discussion

Here, we presented a patient with a rare pathologic entity of the breast as diagnosed with UPS. As suggested in the literature, breast sarcomas are rare, and the breast is also an uncommon site for UPS. Only a few cases have been reported with primary breast UPS. We did a literature review on all previously reported cases of breast UPS, and the data are summarized in Table 1. Such a table may provide more information on the description of the clinical presentation, diagnosis, and treatments used for breast UPS. There were also other cases reported with breast UPS, secondary to cancer treatment (7-13), which we did not include in this table, as we aimed to report the characteristics of the primary breast UPS. In the following, we discuss the most important findings in each case reported. 

**Table 1:** Characteristics of the previously reported cases of breast undifferentiated pleomorphic sarcoma

Sex	Age	Chief complaint	Duration of symptoms	Side	Size	Diagnostic method	Pathological view	Immunohistochemistry	Treatment	Outcome	Reference
M	76	Palpable mass	2 months	Left	1 cm	Needle biopsy	Atypical cells with fibrous proliferation	Not performed/mentioned	Wide excision	Local recurrence after one year	(6)
F	65	Not mentioned	Not mentioned	Left	5 cm	Not mentioned	Not mentioned	Not mentioned	Mastectomy + radiotherapy	Bowel metastasis after 3 years	(14)
F	60	Mass	6 months	Left	6 cm	Fine needle aspiration	Pleomorphic spindle cells with multinucleated tumor giant cells	Not performed/mentioned	Wide excision of the breast without lymph node	Died 3 weeks after	(15)
F	58	Massive swelling + neoplastic fever	3 months	Left	Swollen breast	Incisional biopsy	Giant cells and atypical spindle cells with pleomorphism	Ki-67 30-90%	Total mastectomy	Recurrence in left thoracic wall and axillary	(16)
F	55	Mass and pain	Not mentioned	Right	>5 cm	Incisional biopsy after 6 months	Not mentioned	Partially positive smooth muscle actin and S-100.	Total mastectomy+eribulin mesylate	Died due to respiratory failure after four months	(17)
F	51	Bloody discharge	Not mentioned	Right	9 cm	Breast biopsy, MRI	Highly atypical spindle cells, multiple perivascular aggregates, coagulative necrosis, and mitoses	High Ki67 (70%)	Radical mastectomy + neoadjuvant and adjuvant chemoradiotherapy	Brain and bilateral lung metastasis after 8 months	(18)
F	50	Non-tender mass in the median areolar area	1 month	Right	35 mm	Ultrasound-guided fine needle aspiration	Epithelioid and spindle cells, and osteoclast-like giant cells	Only vimentin was positive	Total mastectomy without axillary lymph node dissection, awaiting histology	No recurrence until 15 months	(19)
F	43	Mass	3 months	Left	3.5	Fine needle aspiration	Oval to spindle-shaped tumor cells with pleomorphism and atypical mitosis	Vimentin and CD68	Modified radical mastectomy	Not mentioned	(20)
F	35	A large fungating and bleeding mass	Not mentioned	Right	17 cm	Fine needle aspiration	High-cellular oval to spindle-shaped tumor cells with pleomorphism and necrosis	Vimentin and CD68	Modified radical mastectomy	Not mentioned	(20)
F	29	Mass	2 months	Left	7 cm	Core biopsy	Spindle cell tumor	Only vimentin was positive	Total mastectomy without lymphadenectomy	Not reported	(21)
F	22	No breast symptoms	2 years	Left	Not mentioned	Not mentioned	Spindle cell tumor	Not mentioned/performed	Mastectomy and adjuvant radiotherapy	Bone metastasis after one year, brain metastasis after two years, death 3 years after breast surgery	(22)

The cases in the table are sorted from the oldest to the youngest to show the effect of age on the outcome of the disease. UPS and sarcomas are generally reported to be diagnosed in the 5^th^ to 7^th^ decades of life (2). Our patient was also 50 years old, similar to other reports available (19). However, data collected and presented in the table show that patients of different ages can have primary breast UPS. This point highlights consideration of this diagnosis regardless of the patient’s age. 

Our patient presented with a fast-growing mass, pain, and pus secretion. The main chief complaint of patients reported in the literature is a non-tender mass (6, 19, 21), while pain and/or pus secretion are rarely reported (16, 18). This implies that the physician should be aware of the range of symptoms a patient with breast UPS may have. An important point to be noted is that breast UPS can also develop in men's breasts. Late diagnosis of UPS and late surgical removal of the breast resulted in local recurrence a year later in a 76-year-old man, presented by Jeong and colleagues (6). Recurrence occurred in this patient despite initial surgery, which emphasizes the significance of negative surgical margins. Fortunately, we performed a total mastectomy in the first step of treatment, and the pathological examination showed free-of-tumor margins. Insufficient evidence is available regarding the need for postoperative treatments, such as chemotherapy, while we performed chemotherapy for the patient to reduce the risk of recurrence or metastasis.

An overview of the patients' outcomes, presented in the table below, shows poor prognosis in many reported cases, possibly related to late- or misdiagnosis. A rare presentation of neoplastic fever, including a 3-month history of general malaise, fever, and weight loss in the case reported by Gambichler and colleagues, with massive swelling, abscess-forming tumor, and pus might have been the cause of late diagnosis, which resulted in recurrence of tumor in the left thoracic wall and axillary, few months after the surgery (16). 

Diagnosis of breast UPS has been also reported in patients who were referred with signs and symptoms of metastasis. Generally, UPS is not a highly metastatic tumor, and lymph node involvement or remote metastasis is rarely reported. Considering the uncertainty in this regard, we excised the sentinel node lymph node during surgery and waited until the pathology was confirmed with no involvement. Some reports have not declared the patient's outcome in their published work (21). Yamazaki and colleagues reported a tumor in the pubic bone with osteolysis and multiple lung metastases at the time the patient's breast UPS was diagnosed; unfortunately, she died four months after the surgery (17). Brain metastasis has also been reported as the presenting symptom (headache, dizziness, and convulsion) in other patients with breast UPS, which resulted in the patient's death after a few months (15, 22). Late referral and not paying attention to the breast tumor the patients had for more than six months might be the cause of the development of a high-grade tumor with metastasis (15). Other cases referred after 1-2 months of mass sensation had better outcomes, like the case presented by Balbi and colleagues, with no recurrence in 15 months of follow-up (19). Even the case reported being treated for the breast lesion before she developed the brain metastasis died after about a year of resecting brain lesions (22). This evidence implies the breast UPS must be excised sufficiently before the patient develops metastasis.

A review of the cases in this study shows that pain may be one of the symptoms, mainly in those patients with metastasis (16, 17); this may suggest the association of pain with the tumor's grade and metastasis, both of which are associated with a poor prognosis. Nipple discharge may also be a factor associated with poor prognosis, as the two cases with this symptom developed metastasis after a few months (16, 18). Similar to other tumors, the tumor size may also be associated with the tumor's grade and the patient's prognosis. However, given the limited cases reported in the literature, there is insufficient evidence for the determination of the prognostic factors in patients with breast UPS, and more studies are required. 

IHC is an efficient method for the differentiation of invasive from non-invasive tumors, especially for challenging breast lesions (23, 24). Therefore, IHC studies would be suggested for breast UPS, as well. We have also tested the IHC of the tumor in our patient, and the results showed positive vimentin and CD68 in the tumoral cells with 50% positivity of Ki67. As shown in the table, some case reports have also performed IHC for the tumor (16, 17, 19-21), while others have not (6, 15, 22). Among those with IHC reports, vimentin positivity seems to be more frequently observed (19-21). But there are also reports of positivity of other markers, like smooth muscle actin and S-100 (17). Vimentin is a significant factor associated with the epithelial-mesenchymal transition in breast tumors, and its positivity is associated with a poorer prognosis, high-grade nuclear cell, and high Ki67 expression in triple-negative (25) and other primary breast cancers (26, 27). In addition, the positivity of other markers, such as Ki67, with vimentin worsens the prognosis (26, 27). A similar association may be present for breast UPS; however, the number of cases reporting IHC results is insufficient to make a definite conclusion.

Further studies may be able to determine the association of vimentin and other IHC markers with the prognosis of patients with breast UPS. Other markers, such as TPRS1, have also been suggested to be highly expressed in spindled-shaped cells and primary breast sarcomas; these markers might help distinguish the type of breast tumor at an initial phase and may improve patients' survival rate by performing a more extensive surgical removal of the tumor (28). In addition, we performed IHC on the resected specimen to confirm the diagnosis. CK was performed to confirm the IHC of the needle biopsy specimen and exclude metaplastic carcinoma. Negative P63 also helped the exclusion of metaplastic carcinoma; furthermore, phyllodes were excluded due to the negativity of the CD34 stain and lack of epithelial components in the microscopic view. Unfortunately, IHC has not been the focus of attention in previous studies and according to the table, IHC studies have not been focused on most of the studies (either on needle biopsy samples or the resected specimen) (6, 15, 22) and only a few studies described IHC studies with a focus on limited markers such as vimentin (16, 17, 19-21). In the present study, we performed several IHCs on needle biopsy and resection samples to establish the diagnosis more accurately.

 The most appropriate diagnostic and therapeutic strategy is also unknown for breast UPS. As mentioned in the table, most of the cases have been diagnosed by needle biopsy sampling of the breast lesion. This implicates the significance of taking an accurate biopsy sample and precise histopathologic examination of the specimens. Treatment of this breast cancer type is also controversial; most have applied excision of the tumor, while some prefer a wide excision or a total mastectomy. Considering the risk of recurrence and metastasis, we suggested adjuvant chemotherapy after total mastectomy. Adjuvant radiotherapy (14, 22) or chemotherapy (18) for patients with breast UPS, has been reported only in a few cases and the most appropriate treatment strategy has remained uncertain and needs to be explored using further cases. Sufficient excision of the primary breast tumor with free-of-tumor margins would be necessary. We suggest the "awaiting histology" approach during surgery for the excision of the lymph nodes. Further studies must determine the most appropriate diagnostic and therapeutic approach for such patients.

## Conclusion

Here, we reported a rare case of breast tumor with a diagnosis of UPS, and we reviewed the previously reported cases in the literature, which has not been described. This type of tumor is more aggressive than other breast sarcomas, which calls for the attention of physicians to consider this rare tumor in mind when approaching a unilateral breast mass. The information on the cases collected and the discussion emphasized in this study can help physicians to improve their knowledge about this tumor. Further studies are required to draw definite conclusions about the characteristics of this tumor and the best diagnostic and therapeutic strategy.

## Funding

This research received no specific grant from any funding agency in the public, commercial, or not-for-profit sectors.

## Ethics Approval:

All procedures performed in this study involving human participants were in accordance with the ethical standards of the institutional and/or national research committee and with the 1964 Helsinki Declaration and its later amendments or comparable ethical standards.

## Informed Consent:

Informed consent was obtained from the patient.

## Conflict of Interest

The authors declare no conflict of interest.

## References

[1] Al-Benna S, Poggemann K, Steinau H-U, Steinstraesser L (2010). Diagnosis and management of primary breast sarcoma. Breast Cancer Res Treat.

[2] Lim SZ, Ong KW, Tan BKT, Selvarajan S, Tan PH (2016). Sarcoma of the breast: an update on a rare entity. J Clin Pathol.

[3] Eilber FC, Kattan MW (2007). Sarcoma nomogram: validation and a model to evaluate impact of therapy. J Am Coll Surg.

[4] Winchester D, Lehman J, Tello T, Chimato N, Hocker T, Kim S (2018). Undifferentiated pleomorphic sarcoma: Factors predictive of adverse outcomes. J Am Acad Dermatol.

[5] Chen S, Huang W, Luo P, Cai W, Yang L, Sun Z (2019). Undifferentiated pleomorphic sarcoma: long-term follow-up from a large institution. Cancer Manag Res..

[6] Jeong YJ, Oh HK, Bong JG (2011). Undifferentiated pleomorphic sarcoma of the male breast causing diagnostic challenges. J Breast Cancer.

[7] Kong J, Shahait AD, Kim S, Choi L (2020). Radiation-induced undifferentiated pleomorphic sarcoma of the breast. BMJ Case Rep.

[8] Rai MP, Mannelli VK, Kandola S, Marinas EB (2017). Pleomorphic sarcoma of the breast. BMJ Case Rep..

[9] Noh JM, Huh SJ, Choi DH, Park W, Nam SJ (2012). Two cases of post-radiation sarcoma after breast cancer treatment. J Breast Cancer.

[10] Komaei I, Guccione F, Sarra F, Palmeri E, Ieni A, Cardia R (2019). Radiation-induced undifferentiated pleomorphic sarcoma of the breast: a rare but serious complication following breast-conserving therapy A case report and literature review. G Chir.

[11] Quadros CA, Vasconcelos A, Andrade R, Ramos RS, Studart E, Nascimento G (2006). Good outcome after neoadjuvant chemotherapy and extended surgical resection for a large radiation-induced high-grade breast sarcoma. Int Semin Surg Oncol..

[12] Early AP, Moon W (2021). Breast cancer and secondary cancer recurrences after autologous tissue reconstruction. Clin Breast Cancer.

[13] Zhang C, Wathuge G (2023). Primary breast sarcoma: a rare and challenging diagnostic entity. Diagn Histopathol.

[14] Cozzolino M, Oliviero C, D'Andrea B, Guglielmi G, Califano G, Caivano R (2018). The role of adjuvant radiotherapy for a case of primary breast sarcoma: a plan comparison between three modern techniques and a review of the literature. Case Rep Med..

[15] Chakrabarti I, Ghosh N, Giri A (2013). Cytologic diagnosis of undifferentiated high grade pleomorphic sarcoma of breast presenting with brain metastasis. J Neurosci Rural Pract.

[16] Gambichler T, Horny K, Mentzel T, Stricker I, Tannapfel A, Scheel CH (2023). Undifferentiated pleomorphic sarcoma of the breast with neoplastic fever: case report and genomic characterization. J Cancer Res Clin Oncol.

[17] Yamazaki H, Shimizu S, Yoshida T, Suganuma N, Yamanaka T, Yamashita T (2018). A case of undifferentiated pleomorphic sarcoma of the breast with lung and bone metastases. Int J Surg Case Rep..

[18] Sang N-V, Duc NM, My T-TT, Ly T-T, Bang LV, Thong PM (2021). A rare case report of breast sarcoma. Radiol Case Rep.

[19] Balbi G, Di Martino L, Pitruzzella G, Pitruzzella D, Grauso F, Napolitano A (2013). Undifferentiated pleomorphic sarcoma with osteoclast-like giant cells of the female breast. World J Surg Oncol..

[20] Bansal A, Kaur M, Dalal V (2017). Pleomorphic sarcoma of breast: a report of two cases and review of literature. Acta Med Iran.

[21] Srinivasamurthy BC, Kulandaivelu AR, Saha K, Saha A (2016). Primary undifferentiated pleomorphic sarcoma of the breast in a young female: a case report. World J Surg Oncol.

[22] Cubas Farinha N, Teixeira W, Roque D, Livraghi S (2022). Large mirror brain metastases from primary undifferentiated sarcoma of the breast: case report and review of the literature. CNS Oncol.

[23] Lee AH (2013). Use of immunohistochemistry in the diagnosis of problematic breast lesions. J Clin Pathol.

[24] Bonacho T, Rodrigues F, Liberal J (2020). Immunohistochemistry for diagnosis and prognosis of breast cancer: a review. Biotech Histochem.

[25] Yamashita N, Tokunaga E, Kitao H, Hisamatsu Y, Taketani K, Akiyoshi S (2013). Vimentin as a poor prognostic factor for triple-negative breast cancer. J Cancer Res Clin Oncol.

[26] Tanaka K, Tokunaga E, Inoue Y, Yamashita N, Saeki H, Okano S (2016). Impact of expression of vimentin and Axl in breast cancer. Clin Breast Cancer.

[27] Hemalatha A, Suresh T, Kumar MH (2013). Expression of vimentin in breast carcinoma, its correlation with Ki67 and other histopathological parameters. Indian J Cancer.

[28] Wang J, Wang W-L, Sun H, Huo L, Wu Y, Chen H (2022). Expression of TRPS1 in phyllodes tumor and sarcoma of the breast. Hum Pathol..

